# Therapeutic Applications of 3D Bioprinting in Surgery for Female Reproductive Tract Disorders: A Systematic Review

**DOI:** 10.3390/polym17223010

**Published:** 2025-11-12

**Authors:** Joaquín Gómez-Abellán, Raquel López-Flores, Juan A. Sánchez-Margallo, Soledad Sánchez-Mateos, Francisco M. Sánchez-Margallo

**Affiliations:** 1 Bioengineering and Health Technologies Unit, Jesús Usón Minimally Invasive Surgery Centre, 10071 Cáceres, Spain; jgomez@ccmijesususon.com; 2 Assisted Reproduction Unit, Jesús Usón Minimally Invasive Surgery Centre, 10071 Cáceres, Spain; rlflores@ccmijesususon.com (R.L.-F.); ssanchez@ccmijesususon.com (S.S.-M.); 3 Red RICORS-TERAV, ISCIII, 28222 Madrid, Spain; msanchez@ccmijesususon.com; 4 Scientific Direction, Jesús Usón Minimally Invasive Surgery Centre, 10071 Cáceres, Spain

**Keywords:** 3D bioprinting, tissue engineering, regenerative medicine, assisted reproduction, female

## Abstract

Three-dimensional printing has been progressively integrated into various industries, particularly the medical sector, where its significance in tissue engineering for transplantation is growing exponentially. The purpose of this systematic review is to ascertain whether the bioprinting of scaffolds holds the potential to provide treatment for pathologies within the female reproductive system. The inclusion criteria applied were the bioimprinting of the ovary, uterus, endometrium, or vagina, intended for surgical implantation in the patient. Articles employing printing methods that do not incorporate cells embedded in the material, those that generate tissue other than that of the female reproductive system, and those that print structures with in vitro applications were excluded from the review. The search for relevant articles was conducted until 3 April 2025. After analyzing 667 articles extracted from PubMed, Scopus and Web of Science, 13 articles were included in this review. The analysis of the results encompassed aspects related to the bioprinting technology employed, the hydrogels and cells utilized, as well as the bioprinted structure and the corresponding target tissue. Few studies investigated the creation of a multicellular scaffold and in none of the cases was it implanted in a large animal model, only in murine and rabbit models. These articles reaffirm the feasibility of employing 3D bioprinting to fabricate tissues and functional organs in the present and future. This advancement will revolutionize the future demand for organs for transplantation.

## 1. Introduction

Considering the characteristics of the different existing technologies, and in order to contextualize this study, 3D bioprinting is hereinafter understood as the process of additive manufacturing with a material composed of living cells suspended in a biomaterial or hydrogel, which in turn may or may not contain biomolecules [1].

The most widely used 3D bioprinting technology today is essentially an adaptation of the Fused Deposition Modeling (FDM) 3D printing technology, patented by Scott Crump in 1989 [2]. It is based on the fusion by temperature of a thermoplastic material that is deposited by extrusion, layer by layer, on a surface by an extruder [3,4].

The first 3D printing technology, Stereolithography (SLA), was invented and patented by Chuck Hull in 1986. In it, the material is generally a photoresin, and is selectively hardened by light, commonly in the ultraviolet spectrum, layer by layer, to form the final model [5]. Odde and Renn were the first to use this technology to deposit live cells in 1999 [6]. There is also the Selective Laser Sintering (SLS) technology patented by Carl Deckard in 1989, in which the material is presented in the form of powder that is fused by a laser layer after layer to shape the model. However, there are currently no applications of this technology in 3D bioprinting [7].

In 1994, researchers at Wake Forest University in North Carolina (USA) were the first to demonstrate that complex structures can be developed from cells using 3D printing. In addition, they were also the first to grow in vitro bladder tissue that was successfully implanted in a patient in 1999 [8], as well as to create a functional experimental organ in 2003, a small kidney capable of secreting urine [9].

The feasibility of bioprinting was first demonstrated in 1988 by Robert J. Klebe, who used a conventional inkjet printer to selectively deposit living cells [10]. The first FDM-type bioprinter on record, which was later marketed as “3D-Bioplotter”, was developed in 2002 by Landers et al. [11].

In the surgical field, the term of xenotransplantation refers to the transplantation of cells, tissues or organs between phylogenetically different species, transplantation when it occurs between phylogenetically identical species [12], and implant, according to the definition of the National Cancer Institute (NCI), is a substance or object placed in the body for therapeutic or diagnostic purposes [13]. Therefore, the use of bioprinting for surgical therapeutic purposes would correspond to an implant.

The growing demand for functional organs for transplantation is evident. The waiting lists for patients to obtain a compatible organ are progressively lengthened over time and xenotransplantation has clear limitations, since not all organs are replaceable and can cause rejection. On the other hand, there are certain ethical implications derived from the use of organs from animals.

The incidence of pathologies affecting the female reproductive system is rising, partly due to increased life expectancy [14]. As far as fertility problems are concerned, difficulties in conception are becoming more and more frequent, either due to follicular depletion with age or the development of various pathologies [15]. For all these reasons, 3D bioprinting with application in tissue engineering can emerge as a highly valuable tool in the pursuit of solutions to these challenges. With its help, functional structures could be created to restore hormonal balance, facilitate embryo implantation, heal wounds or even reconstruct damaged structures. 3D bioprinting raises the possibility of artificially developing personalized solutions that can alleviate the existing demand for therapeutic treatments.

Frances-Herrero et al. conducted a review on reproductive tissue bioengineering in 2022 [16]. While Francés-Herrero’s systematic review published in 2022 provided a broad overview of bioengineering strategies applied to female reproductive organs by extracting data such as article titles, authors, year of publication, targeted organ, engineering approach, materials used, animal species, cellular or tissue models, and experimental setting (in vitro or in/ex vivo), it lacked a focused analysis of 3D bioprinting as a standalone strategy for therapeutic device development. In contrast, the present review addresses this gap by conducting an exhaustive search of studies specifically employing 3D bioprinting to fabricate devices intended for the surgical treatment of reproductive pathologies. This review goes beyond general descriptors by analyzing critical technical parameters, including the type of bioprinter used, bioink composition, crosslinking methods, scaffold architecture, sterilization strategies, degradation studies, cell types and concentrations, animal models employed, and the mechanical and functional outcomes of the printed constructs. This level of detail is essential to assess the translational potential of 3D bioprinting in reproductive medicine and to identify the key challenges and next steps required for clinical application. Among all the collected articles, Frances-Herrero et al. [16] presented only two on endometrial bioprinting [17,18], three on ovarian bioprinting [19,20,21], and four on cervix [22,23,24,25].

Therefore, this review seeks to comprehensively synthesize the advancements in the field of 3D bioprinting of tissues or organs from the female reproductive system with medical applications that have occurred in recent years. This will provide a complete understanding of the current state of the art in tissue engineering. By identifying potential future advancements, we can pave the way for the development of functional reproductive tissue implants through bioprinting. This approach holds the promise of restoring hormonal conditions, fertility, and overall well-being in women.

Specifically, in this systematic review we will analyze the current scientific literature to determine the possibility of creating by bioprinting functional tissues or organs belonging to the female reproductive system to serve as surgical treatment in patients with various pathologies. More specifically, we will mainly focus on the treatment of wounds, hormonal disorders and infertility [26]. Likewise, we will identify: (i) which bioprinting technologies are the most used for this purpose; (ii) what type of analytical and characterization tests are carried out to ensure that the bioprinted structure emulates the mechanical properties of the target tissue; (iii) which hydrogels and cells are the most widely used in bioprinting of tissue belonging to the female reproductive tract; and (iv) we will seek to verify that the cells used not only survive the bioprinting process but are also able to propagate.

## 2. Materials and Methods

As a preliminary step, an exhaustive analysis of the Cochrane Library was performed to identify any potential preexisting systematic reviews that might be relevant [27]. Upon confirming the absence of any comparable reviews, this systematic review was carried out in accordance with the Preferred Reporting Items for Systematic Reviews and Meta-Analyses (PRISMA) guidelines [28]. The research question was formulated using the PICO model for structured question development [29], from which the search string suitable for scientific databases PubMed, Scopus, and Web of Science was designed [30]. The articles retrieved from these databases were imported into the Parsifal platform v2.2 [31], where the refinement of the selection of papers and their peer review was carried out.

### 2.1. Structured Question

The resulting structured PICO question was formulated as follows: “Is it feasible to bioprint scaffolds that replicate the properties of female reproductive tract tissues for surgical implantation as a therapeutic modality to restore hormonal balance, fertility and other pathologies?”.

### 2.2. Search Strategy

The keywords for this review were determined according to the structured question, and the corresponding search string was designed to search for scientific articles categorized in the field of reproductive medicine and bioprinting:

(“Reproductive Techniques” OR “Ovar*” OR “Uter*” OR “Endomet*” OR “Fallopian tub*” OR “Cervix” OR “oviduc*” OR “vagin*” OR “intrauterine adhesions” OR “uterine polyp*” OR “polycystic ovary syndrome” OR “primary ovarian insufficiency*” OR “Female reproductive organs” OR “Female infertility”) AND (“Bioprint*” OR “three-dimensional print*” OR “3D print*” OR “bioink”)

### 2.3. Inclusion and Exclusion Criteria

The study period was adjusted between January 2004 and April 2025. Articles written in a language other than English or Spanish, as well as reviews, were excluded.

All articles that utilized bioprinting for the development of tissues or organs within the female reproductive system for surgical or therapeutic purposes were included. Specifically, studies were considered where 3D bioprinting was employed to develop bioprinted scaffolds or structures that mimicked structures of the female reproductive system. The objective of these structures was to treat associated pathologies by surgical approach, and the proposed solution must be intended for in vivo use.

### 2.4. Classification Method

Articles were categorized based on organs or tissues, into ovary, uterus, endometrium and vagina.

For each study, various aspects were analyzed, such as the bioprinting process details, cell culture information, degradation analysis, and animal model utilization. The synthesis of this information was presented in tabular form, accompanied by percentage figures.

Regarding the bioprinting process, the type of the bioprinter used, the 3D design software used, whether various technologies other than 3D bioprinting had been used in the manufacture of the scaffold, the hydrogel preparation procedure, the hydrogel sterilization method, and whether rheological analyses of the hydrogel or scaffold or scanning electron microscopy (SEM) studies of its microarchitecture had been performed were analyzed.

In the context of the cell culture, the methodology employed for the isolation and characterization was scrutinized. Specifically, it was assessed whether SEM studies were conducted to elucidate the distribution of cells in the scaffold, evaluate their survival rates following bioprinting, and determine their biocompatibility with the hydrogel.

For the degradation aspects, information on scaffold degradation studies conducted both in vitro and in vivo was comprehensively analyzed.

Concerning the animal model, the procedure for creating the animal model of the pathology of interest and the description of the surgical intervention carried out for the implantation of the scaffold were analyzed. Additionally, it was determined whether histological studies of the implant were conducted after the experimental period. Furthermore, the performance of scaffold functionality tests, both in vivo and in vitro, was evaluated, depending on the effect expected to be achieved. Moreover, studies assessing the immune response to the presence of the scaffold and the degree of vascularization or angiogenesis of the scaffold were analyzed.

To assess the methodological quality and risk of bias of the included preclinical studies, we used the Systematic Review Center for Laboratory animal Experimentation (SYRCLE)’s Risk of Bias Tool, which is specifically designed for animal intervention studies [32]. This tool evaluates ten domains related to selection, performance, detection, attrition, reporting, and other potential sources of bias. This information was summarized in a graph for each study included in this review.

Data extraction was performed by a single reviewer. To minimize errors, a subsequent cross-check was performed on the extracted data.

## 3. Results

**Figure 1:**
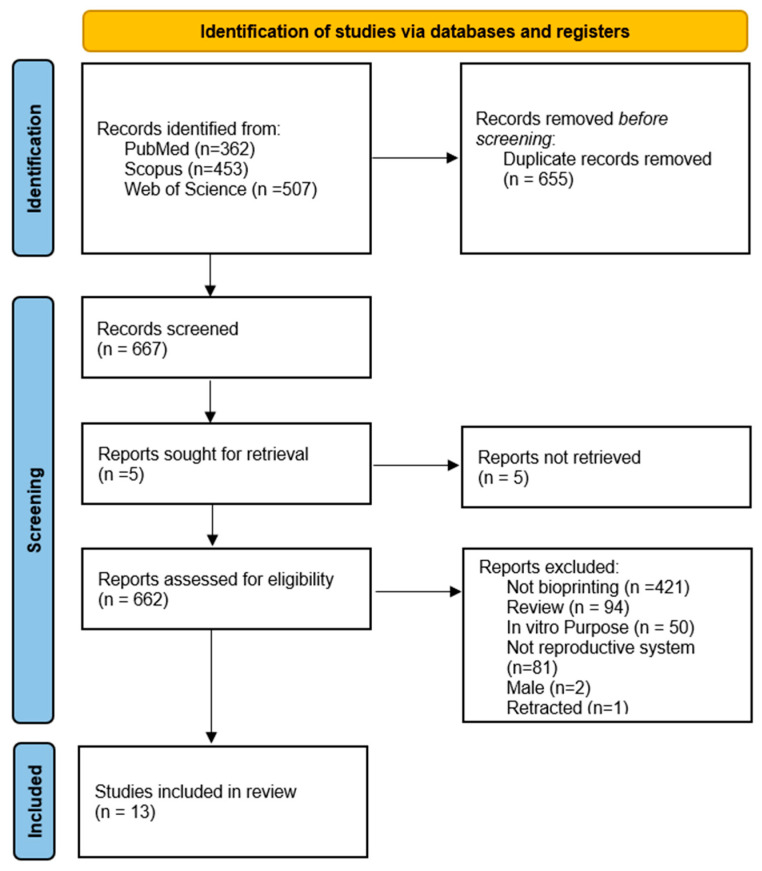
PRISMA flow diagram. All articles included and excluded during article screening are described.

Of the 1322 articles retrieved from the databases analyzed, 655 duplicates were removed. Of the remaining 667 articles, 5 were requested but could not be obtained, leaving 662. Of these, 421 were excluded because they were not related to bioprinting, 94 were review studies, 50 focused on in vitro purposes, 81 were not related to the reproductive system, and one was withdrawn. This resulted in a total of thirteen articles included in this study (Figure 1). A completed PRISMA 2020 checklist is provided in the Supplementary Materials.

To identify potential overlooked articles, a manual citation search was conducted, identifying 914 articles. Duplicates were eliminated, leaving 415 articles for analysis. After analyzing their titles, only 158 articles were selected for further review of their abstracts. Once the abstracts of these papers were analyzed, all the articles considered in this manual analysis phase were ultimately discarded.

Once the articles were classified according to the tissue or organ, the classification groups obtained were ovary, uterus, endometrium, and vagina. Although the fallopian tube also belongs to the female reproductive system, no article on this anatomical structure was identified in this review. As a result, a total of 4 articles dealing with ovarian bioprinting [21,33,34,35], 3 for uterus [36,37,38], 5 for endometrium [18,35,39,40,41] and 2 for vagina were included [23,42].

The included articles fell between 2019 and 2025, with a distribution of 7.7% in 2019, 7.7% in 2020, 15.4% in 2021, 23.1% in 2022, 15.4% in 2023 23.1% in 2024, and finally 7.7% in 2025. Consequently, most of the articles were published in 2022 and 2024.

It is worth noting that most of the articles came from China (61.5%), while the remaining articles were distributed among Hong Kong (15.4%), Korea (7.7%), Australia (7.7%) and Germany (7.7%).

Table 1 presents the results of the systematic review analysis of the studies included in this review, categorized by the respective organs or tissues of the female reproductive system: ovary, uterus, endometrium, and vagina. The extracted information was divided into four primary groups: the equipment employed, the composition and preparation of the hydrogel, the selected cells, and the animal model utilized for in vivo studies.

The analyzed articles reveal that 3D bioprinting holds significant therapeutic potential for a variety of pathologies affecting female reproductive tissues. In the case of ovarian tissue, studies highlight its application in addressing premature ovarian insufficiency (POI), ovarian failure, and fertility decline, particularly in patients undergoing chemotherapy or suffering from ovarian cancer. Uterine tissue bioprinting has been explored as a strategy to treat intrauterine adhesions (IA), absolute uterine factor infertility (AUFI), and general infertility. For endometrial tissue, biofabrication techniques aim to restore functionality in conditions such as IA and pelvic organ prolapse. Vaginal tissue bioprinting, meanwhile, has shown promise in reconstructive approaches for congenital anomalies like Mayer–Rokitansky–Küster–Hauser (MRKH) syndrome, intersex conditions, and vaginal cancer. These advancements suggest that 3D bioprinting could become a transformative tool in regenerative gynecology, offering personalized and fertility-preserving alternatives to conventional treatments.

These studies consistently report high in vitro cell viability in bioprinted reproductive tissue constructs, with survival rates exceeding 85% under the conditions detailed in Table 1. Despite this promising indicator of biocompatibility, functional assessments remain limited. Specifically, only two ovarian tissue studies evaluated hormonal recovery, focusing on estrogen (E2), progesterone (P), and follicle-stimulating hormone (FSH) [33,34]. In both cases, in vivo experiments demonstrated that bioprinted constructs containing ovarian cells were capable of restoring hormone levels in animal models to values closely approximating those of the healthy control group. Regarding embryo implantation success, only one endometrial study conducted a direct evaluation [39]. This study compared implantation rates across three groups: a healthy control, a defect-only group without treatment, and a group with a bioprinted patch applied to a defect site. Results analyzed only embryo implantation that occurred specifically at the defect site. The bioprinted group achieved implantation rates nearly equivalent to the control, while the untreated defect group exhibited less than half the success rate observed in the control. These findings underscore the potential of 3D bioprinting not only to support cellular viability but also to restore key reproductive functions.

Of the eight studies focused on uterine and endometrial bioprinting, only five included rheological assessments to analyze the mechanical properties of the printed constructs in comparison with native uterine tissue [18,36,37,39,40]. These analyses typically measured storage modulus, stretch modulus, and Young’s modulus. Although the reported values were generally close to those of native tissue, none of the constructs achieved mechanical parameters equal to the strength and elasticity of real uterine tissue. Furthermore, while mechanical characterization has been partially addressed, other critical physiological factors remain unexplored in the selected articles. For instance, the high osmotic pressure of follicular fluid in ovarian environments and the low-pH, high-salt microenvironment of the vaginal tract could significantly influence the long-term stability and performance of bioprinted structures. Consequently, it is advisable to undertake supplementary research that incorporates these variables to simulate in vivo conditions more accurately and enhance the reliability and longevity of bioprinted reproductive tissues.

In all instances, the bioprinting technology selected was FDM, utilizing a range of materials for the production of hydrogels. These hydrogels are based on structural proteins found in the extracellular matrix [43], which are employed in variable concentrations. The sterilization process for the hydrogel is achieved through heat treatment or filtration.

The bioprinted structure was a porous mesh with a circular or square base, occasionally multilayered through the utilization of additional technologies such as casting or electrospinning. While there is a discernible trend towards the utilization of cells from various sources of the reproductive system, it is also possible to find cells from other tissues, such as mesenchymal cells from adipose tissue or bone marrow, at a concentration of approximately 106 cells/mL.

Synthetic materials or materials derived from algae are also incorporated to optimize the crosslinking process. This crosslinking is primarily achieved through UV light exposure for a few seconds or by adding CaCl_2_ for a maximum of five minutes.

In vivo studies were performed in a murine animal model and, on one occasion, in a rabbit model. Only five of the thirteen articles described the study of the degradation of the construct, typically conducted in vitro.

**Figure 2:**
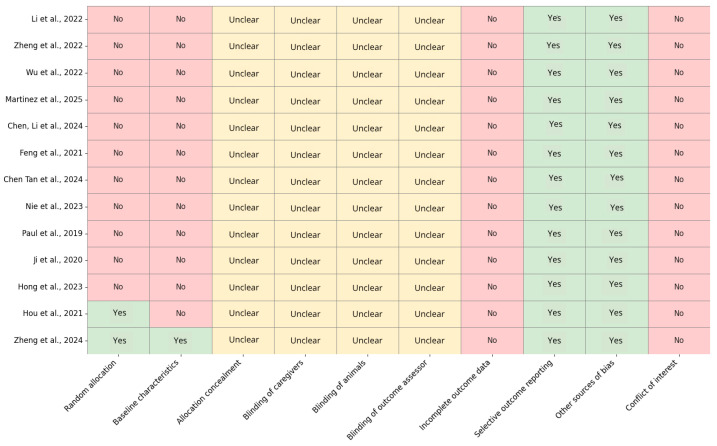
Bias risk analysis using the SYRCLE methodology is summarized in the following table. Each item is rated as ’Yes’ (indicating low risk of bias), ’No’ (high risk of bias), or ’Unclear’ (insufficient information to determine the risk). Studies analyzed: Li et al., 2022 [33]; Zheng et al., 2022 [34]; Wu et al., 2022 [21]; Martinez et al., 2025 [35]; Chen, Li et al., 2024 [36]; Feng et al., 2021 [37]; Chen Tan et al., 2024 [38]; Nie et al., 2023 [39]; Paul et al., 2019 [40]; Ji et al., 2020 [18]; Hong et al., 2023 [41]; Hou et al., 2021 [23]; and Zheng et al., 2024 [42].

To evaluate the risk of bias in the 13 preclinical studies included, the SYRCLE’s Risk of Bias Tool was used (Figure 2). The analysis indicated a high or unclear risk of bias in the majority of the assessed domains. Notably, only two studies [23,42] explicitly reported random allocation, while the remaining studies failed to do so, potentially suggesting a selection bias. Furthermore, baseline characteristic balance was poorly reported, with only one additional study meeting this criterion. Regarding allocation concealment and the varying levels of blinding (caregivers, animals, and outcome assessors), all studies lacked sufficient information and were classified as “unclear,” indicating a systematic deficiency in methodological transparency. The risk of bias attributable to incomplete outcome data was low across all studies, which is a positive outcome. However, selective outcome reporting and other sources of bias were frequently observed, although most studies exhibited a low risk in these domains.

## 4. Discussion

Bioprinting holds transformative potential for surgical therapies of the female reproductive tract by enabling the creation of patient-specific bioengineered tissues that closely resemble native anatomical structures. This technology facilitates the development of customized artificial tissue for vaginal reconstruction, uterine repair, and ovarian tissue engineering, thereby enhancing outcomes in cases of congenital anomalies, oncologic resections, or infertility. By employing biocompatible materials and incorporating living cells, bioprinted constructs promote tissue integration and functional regeneration, thereby mitigating the complications associated with conventional surgical implants. As research progresses, bioprinting is anticipated to assume a pivotal role in personalized and regenerative gynecologic surgery. In this systematic review, a comprehensive analysis of current advancements in the application of 3D bioprinting for surgical therapeutic enhancements in the treatment of female reproductive tract pathologies has been undertaken to provide insights into bioprinting techniques, their associated technology, and the materials utilized.

These functional structures, known as scaffolds, have shown promising results in various animal models [18]. For instance, in a murine model, scaffolds have been successfully integrated by the target tissue, facilitating embryo implantation in the uterus and endometrium, or significantly restoring hormone production levels in the ovary [33]. Additionally, scaffolds have demonstrated the potential to promote the regeneration and endothelization of vaginal wounds [42].

Nevertheless, it is crucial to acknowledge that the development of bioprinted scaffolds capable of replacing structurally intricate organs with diverse integrated cell types will likely necessitate several years of research and development. One potential solution lies in the combined use of various tissue engineering techniques, such as electrospinning, molding, coating, and bioprinting. These techniques have been successfully employed in articles related to bioprinting the uterus [36,38], but their functionality has not been demonstrated in animal models.

Successful bioprinting is a multifaceted process that necessitates careful consideration of numerous factors. Key aspects include the bioprinter’s technology, the chosen materials, the desired structure, the sterilization method, and the crosslinking process. In light of the primary findings derived from this systematic review, we will proceed to analyze each of these factors in detail.

In all articles analyzed in this review, the bioprinting technology employed was FDM. Notably, none of the articles utilized SLA technology, which offers superior precision. This may be attributed to the fact that these printers operate based on the exposure of the bioink to ultraviolet light during the manufacturing process. Given the variety of bioprinters employed across the articles, it is not feasible to suggest a particular equipment model. Nevertheless, we can provide insights into the essential characteristics that a bioprinter should incorporate for its optimal utilization in 3D bioprinting applications for the treatment of female reproductive system pathologies with potential clinical impact, including uterine fibroids, endometriosis, ovarian cysts, endometrial and cervical polyps, and polycystic ovary syndrome.

The hydrogel should be a biocompatible medium that can be extruded without damaging the cells under the necessary pressure. Additionally, it must have the property of cross-linking by means of a non-cytotoxic treatment. The most frequently chosen materials for hydrogel formulation include gelatin [18,21,23,33,34,36,37,38,42], sodium alginate [18,21,23,33,34,35,39,40,41], and decellularized extracellular matrix [33,34,35,42]. These materials are structural proteins that create a three-dimensional network, providing a biocompatible environment similar to that found in the organism. They facilitate interactions that enable cells to fulfill all their biological functions. In most cases, multiple materials are used in hydrogel production. This is because, while a material may possess excellent bioprinting properties, it may not necessarily cross-link or provide a favorable environment for cells.

In most articles, the structure chosen for bioprinting was a mesh, designed to facilitate the integration of the structure into the target tissue and promote effective neoangiogenesis. Typically, this mesh is constructed using the same hydrogel and a single cell type. However, three articles presented alternative approaches. Nie et al. fabricated a bilayer scaffold by molding a disc with a single cell type and bioprinting a mesh with a second cell type on top, using the same hydrogel [39]. In contrast, Paul et al. developed a polycaprolactone (PCL) mesh created by melt electrospinning (MES) on which an Alg and AV hydrogel was bioprinted with eMSCs [40]. Similarly, the studies of Li et al. and Chen, Tan et al. were focused on creating shape-memory trilayered uterine scaffolds [33,38]. The first study constructed a first layer of shape memory poly (L-lactide-co-trimethylene carbonate) (PTMC) and polyurethane (TPU) on which they electrospun a second layer composed of poly (lactic acid-co-glycolic acid) (PLGA), GelMa, polydopamine (PDA) and estradiol (E2) [33]. Finally, they bioprinted a hydrogel of GelMa and Gel with embedded BMSCs on the bilayer. The second study aimed to fabricate a similar structure using different technologies [34]. The first shape memory layer was molded from PTMC and PLGA, the second layer was applied by coating, depositing PDA, E2 and HA, and the third layer was bioprinted with the same GelMa, Gel and BMSCs hydrogel as in the first study.

In cases where the hydrogel used to suspend cells during bioprinting cannot emulate tissue’s mechanical properties, some studies employed alternative strategies such as 4D printing [44], electrospinning [45], molding [46], and coating [47]. These techniques collectively enabled the creation of complex structures with diverse materials and functionalities.

Given that the purpose of a bioprinted scaffold is surgical implantation, it is imperative to maintain optimal sterile conditions throughout its development. This can be achieved by adhering to all steps, from hydrogel development to the final completion of the bioprinted scaffold, under sterile conditions. In the absence of necessary sterilization methods, measures must be implemented to ensure the sterility of the hydrogel, as mixing it with cells would render any subsequent sterilization process ineffective.

**Figure 3:**
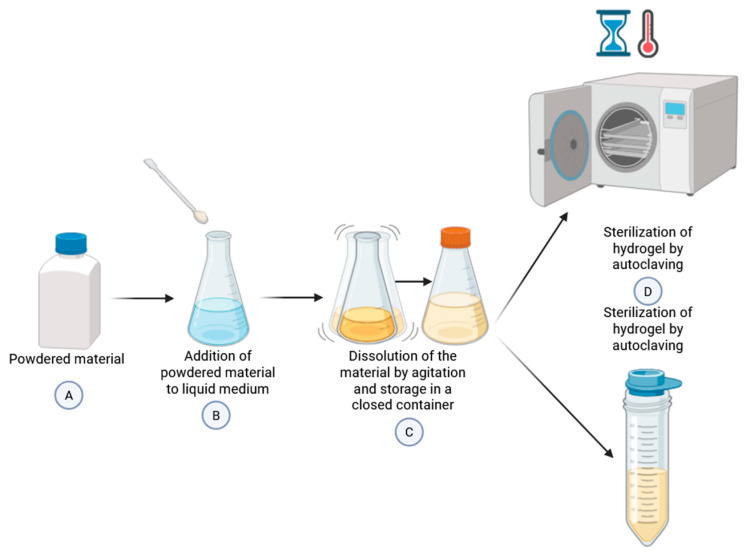
Process for preparing and sterilizing hydrogel for use in bioprinting.

The articles referenced indicated that the most prevalent sterilization methods involve subjecting the hydrogel to multiple high-temperature cycles, effectively eliminating microorganisms (autoclave) [21,23,33,42]. However, this method may not be suitable for heat-hardening hydrogels. Filter sterilization was also employed [21], which involves passing the hydrogel through a filter capable of retaining microorganisms while allowing the hydrogel to pass through. For illustration, Figure 3 presents a diagram depicting the conventional manufacturing and sterilization process of hydrogel, from the mixing of materials to the addition of cells.

Generally, all hydrogels used in bioprinting undergo crosslinking treatment to preserve their structure without disintegration. This process typically involves chemical treatment for a specific duration, often employing calcium chloride [18,23,33,34,35,39,41], or exposure to an ultraviolet (UV) light source emitting at approximately 405 nm at a designated distance [21,36,37,38,42]. Contrary to the assumption that UV light exposure to cells would be detrimental, five out of thirteen studies demonstrated favorable cell survival rates when employing materials that require such treatment [21,36,37,38,42]. 

Among the current limitations of 3D bioprinting for female reproductive system therapies, this review emphasizes the technology’s inability to construct intricate and functional organs. Advancements in technology have enabled the printing of basic functional tissues, yet the development of multicomponent organs remains an arduous endeavor that necessitates several years of dedicated research. Conversely, the absence of substantial studies demonstrating results in large animal models restricts their translational validity.

A notable limitation across the analyzed studies is the absence of direct comparisons between 3D bioprinted constructs and traditional grafting techniques, such as autologous tissue transplantation, cryopreserved allogeneic ovarian grafts, and uterine transplantation. While the in vitro fabrication of bioprinted devices offers clear advantages, particularly by eliminating the need for donor tissue from patients or animals, the lack of benchmarking against established clinical approaches hinders a comprehensive evaluation of their therapeutic efficacy. Comparative studies would be highly valuable to determine whether bioprinted constructs can match or surpass the functional outcomes of conventional treatments, especially in terms of tissue integration, hormonal restoration, and reproductive success. Such investigations are essential to validate the clinical relevance of 3D bioprinting and to guide its translation into routine gynecological practice.

The findings of this review indicate that while 3D bioprinting holds promise as an increasingly utilized technology in the field of tissue engineering, its application in the treatment of reproductive system pathologies remains limited. Therefore, it is of paramount importance to compile a comprehensive compilation of all extant literature to ascertain the cell lines, biomaterials, and bioprinting technologies employed. This compilation will facilitate the identification of the most promising future avenues for tissue engineering, enabling the development of functionalized tissues and organs that can be successfully implanted. 

The risk of bias assessment conducted using the SYRCLE’s Risk of Bias Tool revealed significant methodological shortcomings across the included preclinical studies. The majority of studies exhibited either substantial or unclear risk in domains pertaining to randomization and blinding, indicating potential threats to internal validity. Notably, only Hou et al. [23] and Zheng et al. [42] reported adequate random allocation, while baseline comparability was exclusively confirmed in Zheng et al. [42]. Conversely, allocation concealment and blinding of caregivers, animals, and outcome assessors were consistently unreported, underscoring a systematic lack of transparency. In contrast, all studies demonstrated low risk for incomplete outcome data, and selective reporting was generally well-managed. No conflicts of interest were declared, although this should be interpreted with caution given the limited reporting detail. Overall, these findings emphasize the imperative for enhanced methodological rigor and reporting standards in animal research to augment the reproducibility and reliability of preclinical evidence.

## 5. Conclusions

Pathologies within the female reproductive system, such as ovarian cancer, endometriosis, and intrauterine adhesions, present substantial medical challenges. These conditions impact fertility, hormone production, and overall well-being. Some, like ovarian cancer, often require the removal of the ovaries, leading to symptoms resulting from the loss of hormone production essential for proper bodily functioning and subsequent complications. Others, such as endometriosis and intrauterine adhesions, cause fertility issues and even pain and discomfort. 

3D bioprinting shows promise in creating functional artificial tissues, or scaffolds, for tissue restoration and enhancement. While scaffolds have demonstrated success in animal models, replacing complex organs with integrated cell types will require further research and development.

While bioprinting functional tissues is currently feasible, it remains challenging to create functional complex organs, particularly those of the female reproductive system. Among the most commonly used 3D bioprinting technologies, Fused Deposition Modeling (FDM) stands out. Hydrogels, particularly gelatin, alginate, or their combinations, are frequently employed as the base material. In many instances, decellularized extracellular matrix (dECM) is also incorporated. Mesenchymal stem cells from various origins are the most commonly used cells in these applications. The typical structure of the artificial tissue is a mesh, which facilitates tissue integration and angiogenesis. Complementary techniques such as electrospinning, molding, coating, and bioprinting are employed, particularly for more intricate structures like the uterus. 

Clinical applications of these techniques include developing media for embryo implantation in the uterus and endometrium, partially restoring hormonal function in the ovary, and regenerating and reendothelializing wounds in the vagina. To assess the functionality of the bioprinted scaffolds, various tests are conducted. These include cross-sections and monitoring of fetal implantation and development, histological tests to check cell occupancy, and evaluation of neoangiogenesis. It would be beneficial to include scaffold degradation tests in future research studies. These tests would help determine the degradation and integration dynamics of the implanted materials and identify potential side effects. 

## Figures and Tables

**Table 1:** Results of the studies analyzed organized by the equipment used, the composition and preparation of the hydrogel, the cells selected and the animal model on which the in vivo studies were performed.

Organ	Bioprinter	Hydrogel	Scaffold	Sterilization	Crosslinking	Degradation	Cells	Cell Concentration (mL^−1^)	Animal Model	Reference
Ovary	Bio-Architect-WS	Gel + Alg + dECM	Cylindrical mesh	Temperature	CaCl^2^	No	ADSCs	1 × 10^7^	Rat	Li et al., 2022 [33]
					5 min					
	Bio-Architect-WS	Gel + Alg + dECM	Cylindrical mesh	-	CaCl^2^	No	POCs	1 × 10^6^	Mice	Zheng et al., 2022 [34]
					3 min					
	Sunp Biomaker 2	GelMa + Alg	Mesh	Temperature and filtration	UV	In vitro	COCs	2 × 10^6^	-	Wu et al., 2022 [21]
	BioX	Alg + dECM	Cylindrical mesh	-	CaCl^2^ + Thrombin 15 min	No	eSCs	1 × 10^6^	No	Martinez et al., 2025 [35]
Uterus	No	GelMa	Trilayer mesh	No	UV	No	BMSCs	1 × 10^6^	No	Chen, Li, et al., 2024 [36]
	EFL-BP-6800	GelMa/ColMa	Mesh	No	Blue light 10 s	In vitro	hAMSCs	No	Rat	Feng et al., 2021 [37]
	regenHU	GelMa/Gel	Trilayer mesh	No	UV	In vitro	BMSCs	1 × 10^6^	No	Chen, Tan, et al., 2024 [38]
Endometrium	Regenovo	Alg + HA	Bilayer mesh	No	CaCl^2^	In vitro/in vivo	EECs/ESCs	1 × 10^6^	Rat	Nie et al., 2023 [39]
	Bioscaffolder	Alg + AV	Bilayer mesh	No	Not required	In vitro	eMSCs	1 × 10^6^	Mice	Paul et al., 2019 [40]
	Tissform III	Gel + Alg	Cylindrical mesh	No	CaCl^2^ 5 min	No	hiMSCs	1 × 10^6^	Rat	Ji et al., 2020 [18]
	Root, Baobab healthcare	Alg + Col/Plu	Hollow cylinder	No	CaCl^2^	No	ADSCs	5 × 10^5^	Rat	Hong et al., 2023 [41]
	BioX	Alg + dECM	Cylindrical mesh	-	CaCl^2^ + Thrombin 15 min	No	eSCs	1 × 10^6^	No	Martinez et al., 2025 [35]
Vagina	Bio-Architect-WS	Gel + Alg + AVM	Cylindrical mesh	Temperature	CaCl^2^	No	BMSCs	1 × 10^6^	Rat	Hou et al., 2021 [23]
	Bio-Architect-WS	ECM + GelMA + SF	Cylindrical mesh	Temperature	UV	No	BMSCs	4 × 10^6^	Rabbit	Zheng et al., 2024 [42]

ADSCs: Adipose derived stem cells; Alg: Alginate; AV: Aloe vera; AVM: Acellular vagina matrix; BMSCs: Bone marrow mesenchymal stem cells; COCs: Cumulus oocyte complexes; Col: Collagen; ColMa: Collagen methacrylate; dECM: Decellularized extracellular matrix; ECM: Extracellular matrix; EECs: Endometrial epithelial cells; ESCs: Embryonic stem cells; eSCs: endometrial stromal cells; Gel: Gelatine; GelMa: Gelatin methacrylate; HA: Hyaluronic acid; hAMSCs: Human amniotic mesenchymal stem cells; hiMSCs: Human induced mesenchymal stem cells; POCs: Primary ovarian cells; Plu: Pluronics; SF: Silk fibroin; UV: Ultraviolet.

## Data Availability

The data are not publicly available due to privacy.

## Supplementary Materials

The following supporting information can be downloaded at: https://www.mdpi.com/article/10.3390/polym17223010/s1: PRISMA checklist.

## Abbreviations

The following abbreviations are used in this manuscript:

ADSCs	Adipose derived stem cells
Alg	Alginate
AV	Aloe vera
AVM	Acellular vagina matrix
BMSCs	Bone marrow stem cells
COCs	Cumulus oocyte complexes
Col	Collagen
ColMa	Collagen methacrylate
dECM	Decellularized extracellular matrix
ECM	Extracellular matrix
EECs	Endometrial epithelial cells
ESCs	Embryonic stem cells
E2	Estradiol
FDM	Fused Deposition Modeling
Gel	Gelatine
GelMa	Gelatin methacrylate
HA	Hyaluronic acid
hAMSCs	Human amniotic mesenchymal stem cells
hiMSCs	Human induced mesenchymal stem cells
MES	Melt electrospinning
NCI	National Cancer Institute
PCL	Polycaprolactone
PDA	Polydopamine
PICO	Population, Intervention, Comparison and Outcome
PLGA	Poly(lactid acid-co-glycolic acid)
Plu	Pluronics
POCs	Primary ovarian cells
PRISMA	Preferred Reporting Items for Systematic reviews and Meta-Analyses
PTMC	Poly (L-lactide-co-trimethylene carbonate)
SEM	Scanning Electron Microscopy
SF	Silk fibroin
SLA	Stereolithography
SLS	Selective Laser Sintering
TPU	Polyurethane
USA	United States of America
UV	Ultraviolet
